# Periostin contributes to the maturation and shape retention of tissue-engineered cartilage

**DOI:** 10.1038/s41598-018-29228-6

**Published:** 2018-07-25

**Authors:** Ryoko Inaki, Yuko Fujihara, Akira Kudo, Masaki Misawa, Atsuhiko Hikita, Tsuyoshi Takato, Kazuto Hoshi

**Affiliations:** 1 Department of Oral-maxillofacial Surgery, Dentistry and Orthodontics, The University of Tokyo Hospital, Tokyo, Japan; 2 Division of Tissue Engineering, The University of Tokyo Hospital, Tokyo, Japan; 3 Department of Biological Information, Tokyo Institute of Technology, Tokyo, Japan; 4 Institute of Human Science and Biomedical Engineering, National Institute of Advanced Industrial Science and Technology, Tsukuba, Ibaraki, Japan; 5 JR Tokyo General Hospital, Tokyo, Japan

## Abstract

Traditional tissue-engineered cartilage applied in clinical practice consists of cell suspensions or gel-form materials for which it is difficult to maintain their shapes. Although biodegradable polymer scaffolds are used for shape retention, deformation after transplantation can occur. Here, we showed that periostin (PN), which is abundantly expressed in fibrous tissues, contributes to the maturation and shape retention of tissue-engineered cartilage through conformational changes in collagen molecules. The tissue-engineered cartilage transplanted in an environment lacking PN exhibited irregular shapes, while transplants originating from chondrocytes lacking PN showed limited regeneration. In the *in vitro* assay, PN added to the culture medium of chondrocytes failed to show any effects, while the 3D culture embedded within the collagen gel premixed with PN (10 μg/mL) enhanced chondrogenesis. The PN-mediated collagen structure enhanced the mechanical strength of the surrounding fibrous tissues and activated chondrocyte extracellular signaling by interstitial fibrous tissues.

## Introduction

Tissue engineering is a new treatment alternative to conventional tissue transplantation. By applying biodegradable polymers as scaffolds, engineered tissues can be freely arranged in shapes. In cartilage regenerative medicine, the reproduction of the 3D shape is particularly important^1,2^.

Cartilage regenerative medicine, including autologous chondrocyte implantation (ACI), has been used in the treatment of cartilage in a knee joint^3,4^. In ACI, autologous chondrocytes obtained from a healthy cartilage site are transplanted into damaged sites. For facial areas, regenerative treatments based on ACI have been indicated for nasal augmentation^5^. Autologous chondrocytes in the gelatinous chondroid matrix are injected into subcutaneous pockets in the nose. However, chondrocytes suspended in solution or gel have difficulty maintaining the structural integrity of engineered cartilage. To overcome this issue, our group has developed tissue-engineered cartilage consisting of autologous chondrocytes and biodegradable polymer scaffolds^6–8^. We have conducted clinical research on patients with nasal deformities associated with cleft lips and palates (JPRN-UMIN000005472). Shape retention is an important consideration for tissue-engineered cartilage in facial areas. Deterioration in the supply of nutrition or oxygen causing insufficient cartilage regeneration^9,10^, the secretion of catabolic enzymes from migrated inflammatory cells^11^, or excessive physical stress applied from an external environment will cause deformity of the transplants^12^. These events occur at the interface between a host and a transplant. Therefore, controlling for these events in the interfacial structure will be essential for retaining the suitable shape of a transplant. During the maturation of regenerated cartilage *in vivo*, fibrous tissues surround the transplants and constitute interstitial areas inside the transplants, both of which can provide a suitable environment for tissue regeneration^13,14^. These fibrous tissues consist of not only collagen but also various proteins, including matricellular proteins, proteoglycans and glycoproteins. The complex of these proteins can support collagen structuring and provide structural properties to collagen-based tissues. We focused on periostin (PN), which is a matricellular protein that is specifically localized in overloaded collagen-based fibrous tissue, such as the periosteum^15^, periodontal ligament^15,16^, and heart valves^17,18^. In addition, PN is prominently upregulated during extracellular matrix (ECM) remodeling, after myocardial infarction^17,18^, in bone marrow fibrosis and in subepithelial fibrosis in bronchial asthma^19^. PN can directly bind many ECM proteins such as collagen, fibronectin, and tenascin-C. We hypothesized that PN expressed in surrounding and interstitial fibrous tissues could promote cartilage regeneration and *in vivo* maturation by improving the collagen structure in both fibrous tissues.

## Results

### Localization of PN in tissue-engineered cartilage

**Figure 1:**
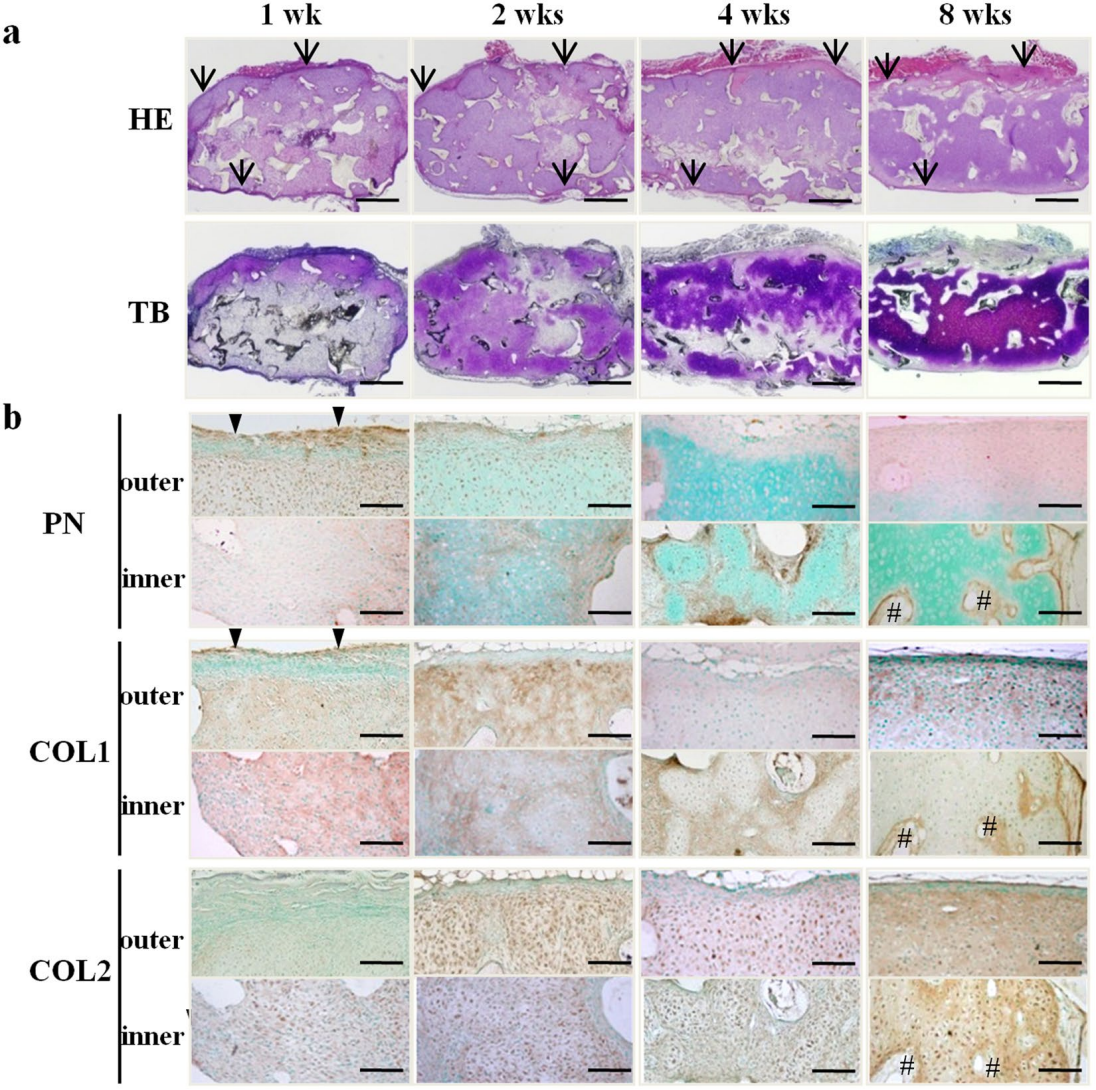
Localization of periostin in tissue-engineered cartilage. Histological analysis of human tissue-engineered cartilage constructs in nude mice up to 8 weeks after transplantation. (**a**) HE and Toluidine blue (TB) staining. Eosinophilic tissues surrounding the constructs were observed from one to 8 weeks (arrow). (**b**) Immunohistochemical staining for PN and type I, or II collagen. PN was expressed in the surrounding tissue at 1 week (arrowheads) and interstitial tissue at 8 weeks (number signs). Scale bars: 1 mm (TB, HE) and 200 µm (other).

We made the tissue-engineered cartilages using a poly-L-lactic acid (PLLA) scaffold and human auricular chondrocytes, and subcutaneously transplanted them into nude mice. The transplants gradually regenerated the cartilage (Fig. 1a), while surrounding fibrous tissues were formed starting at 1 week (Fig. 1a arrows). In this surrounding area, PN was co-localized with type I collagen (COL1) at 1 week (Fig. 1a arrowheads). The interstitial fibrous tissues that intervened among the cartilage regenerating areas were positive for PN from 2 weeks to 8 weeks, and the PN also co-localized with COL1 but not with type II collagen (COL2) (Fig. 1a number sign). Next, we performed real-time PCR (RT-PCR) analysis using specific primers for human (transplant) or mouse (host) genes. In the transplantation of the tissue-engineered cartilage (Fig. S1 AC + PLLA), the expression of *mCol1a1* and *mPeriostin* was observed at 1 week, in contrast to that of *hCOL1A1* and *hPERIOSTIN*, which was detected during the later stages of transplantation (2 and 8 weeks). The timing of PN expression was different between mice and humans. These results suggested that PN was expressed in both the host and the transplant.

### The functions of host-derived PN and transplant-derived PN

**Figure 2:**
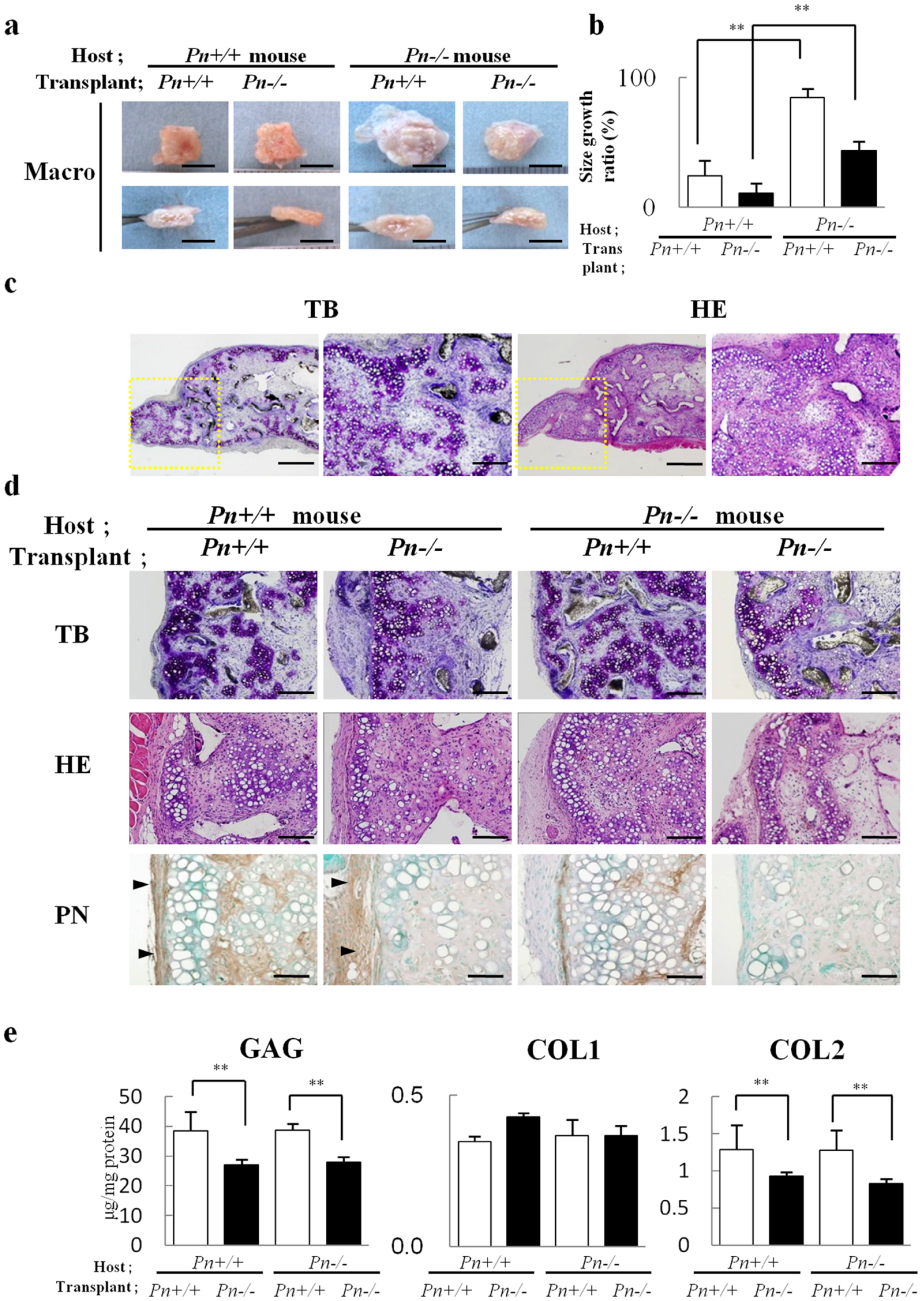
Transplantation of tissue-engineered cartilages into *Pn*+/+ or *Pn*−/− mice. Tissue-engineered cartilage constructs consisting of *Pn*+/+ or *Pn*−*/*− auricular chondrocytes (Transplant) were transplanted into *Pn*+/+ or *Pn*−/− mice (Host). Eight weeks after transplantation, the constructs were analyzed. (**a**,**b**) The morphological features and the size growth ratio of the constructs. The areas of the constructs were analyzed from the photographs using ImageJ software. The size growth ratio was calculated from the ratio of areas of regenerated cartilage extending out of PLLA scaffolds to those of original areas. Scale bars, 5 mm (**a**). **P < 0.01, vs Host; *Pn*+/+ mice (**b**). (**c**) HE and TB staining in a construct containing the *Pn*+/+ chondrocytes (Transplant; *Pn*+/+) transplanted into *Pn*−/− mice (Host; *Pn*−/− mouse). Magnified images of overflow sites (dotted line regions) showed the construction of cartilage tissues. Scale bars, 1 mm (left), 500 µm (right) (HE and TB). (**d**) HE, TB staining and immunohistochemical staining for PN. PN was observed in the interstitial area in the constructs containing the *Pn*+/+ chondrocytes (Transplant; *Pn*+/+) and in the surrounding areas in the constructs transplanted into *Pn*+/+ mice (Host; *Pn*+/+ mouse, arrowheads). Scale bars: 500 µm (TB), 200 µm (HE), and 100 µm (PN). (**e**) The contents of GAG, COL1 and COL2 were analyzed. Means (bars) ± SEM (error bars) for 3 implants/group. **P < 0.01, vs Transplant; *Pn*+/+.

To investigate the effect of PN in these locations, we transplanted the tissue-engineered cartilage using chondrocytes derived from *periostin*+/+ (*Pn*+/+) and *periostin*−/− (*Pn*−/−) mice into the subcutaneous pockets of each mouse. Regardless of the origin of the chondrocytes, the transplants into the *Pn*+/+ mice maintained their 3D morphology at 8 weeks (Fig. 2a Host: *Pn*+/+ mice), while the transplants into the *Pn*−/− mice exhibited irregular shapes (Fig. 2a Host: *Pn*−/− mice), which extended considerably out of the original shapes (Fig. 2b). In transplants (Host: *Pn*−/− mice, Transplant: *Pn*+/+ mice) which showed the most morphological irregularities, histological findings showed that the site overflowing from the scaffold consisted of regenerated cartilage tissue (Fig. 2c).

Histologically, the transplants into the *Pn*−/− mice were devoid of PN in the surrounding fibrous tissues (Fig. 2d Host: *Pn*−/− mice). Transplants containing the *Pn*−/− chondrocytes (Fig. 2d Transplant: *Pn*−/−), in which the interstitial fibrous tissues showed no localization of PN showed suppression of chondrogenesis. The amount of glycosaminoglycan (GAG) and COL2 were decreased in transplants containing *Pn*−/− chondrocytes compared to transplants with *Pn*+/+ chondrocytes (Fig. 2e). These results suggested that the host-derived PN in the surrounding fibrous tissues contributed to the maintenance of the transplants shape, and that the transplant-derived PN in the interstitial fibrous tissues supported the formation of the cartilage matrices. Despite the different functions of the host- and transplant-derived PNs, they were similarly co-localized with COL1.

### Involvement of the EMI domain in the interaction between PN and COL1

**Figure 3:**
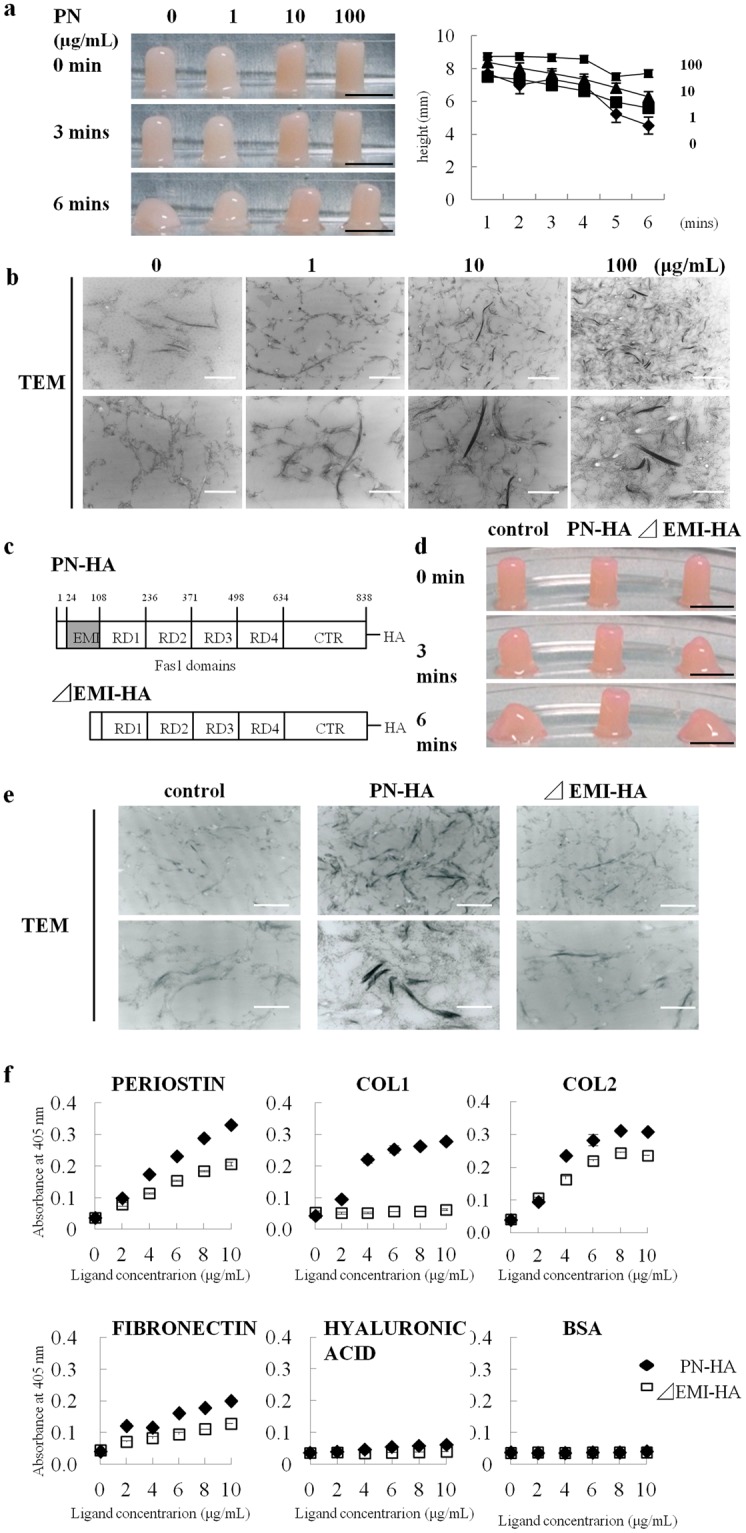
Interaction between the EMI domain of PN and type I collagen. (**a**) Morphological features of gelled collagen with PN (1, 10, 100 µg/mL) or without PN (0 µg/mL) with time. (**b**) TEM images of gelled collagen. PN addition improved the organization of the collagen- network in a dose dependent manner. (**c**) Schematic view of the EMI domain deletion forming (*Δ*EMI-HA) and no- deletion forming PN (PN-HA). (**d**,**e**) Morphological features and TEM images of gelled collagen in PN-HA (10 µg/mL) and *Δ*EMI-HA (10 µg/mL). (**f**) The binding assay of PN to the other ECM protein. Binding ability is indicated by the concentration of biotinylated PN-HA (closed square) and *Δ*EMI-HA (open square). BSA (bovine serum albumin) was used as a control. Scale bars for (**a**) and (**d**): 1 cm, and for (**b**) and (**e**): 250 nm (upper), and 100 nm (lower).

We next examined the interaction between PN and COL1 using a recombinant PN and collagen gels. When the gel formation of the collagen following PN addition was compared to that without PN, the addition of PN enabled the collagen gel to maintain its height in a dose-dependent manner (Fig. 3a). Transmission electron microscopy observations revealed that PN promoted the association of the collagen molecules, forming the assembled fibers (Fig. 3b). PN consists of six domains: the N terminal EMI domain, four tandem repeats of the FAS1 domain, and a C terminal region (CTR). Previous studies suggested that the EMI domain could bind and interact with fibronectin and COL1^12,14^. However, the direct interaction between PN and COL1 remained to be elucidated. To confirm the binding domain of PN to COL1, we compared wild-type PN (PN-HA) and a deletion form of PN with the EMI domain deletion (*Δ*EMI-HA) (Fig. 3c). The addition of *Δ*EMI-HA could not maintain the columnar shape of the collagen gel, while the addition of PN-HA could maintain the columnar shape (Fig. 3d). In the transmission electron microscopy image, PN-HA promoted the formation of the assembled collagen fibers, while *Δ*EMI-HA failed to form the gathering fibers and induce collagen conformation (Fig. 3e). To further identify the binding state of PN, we performed an adhesion assay with other matrix proteins. *Δ*EMI-HA suppressed the binding of wild-type PN and COL1, although it did not affect binding with COL2, fibronectin, hyaluronic acid, or bovine serum albumin (BSA) (Fig. 3f). These results indicated that the EMI domain played an important role in the interaction with COL1.

### The effect of PN-premixed collagen on chondrocyte differentiation

**Figure 4:**
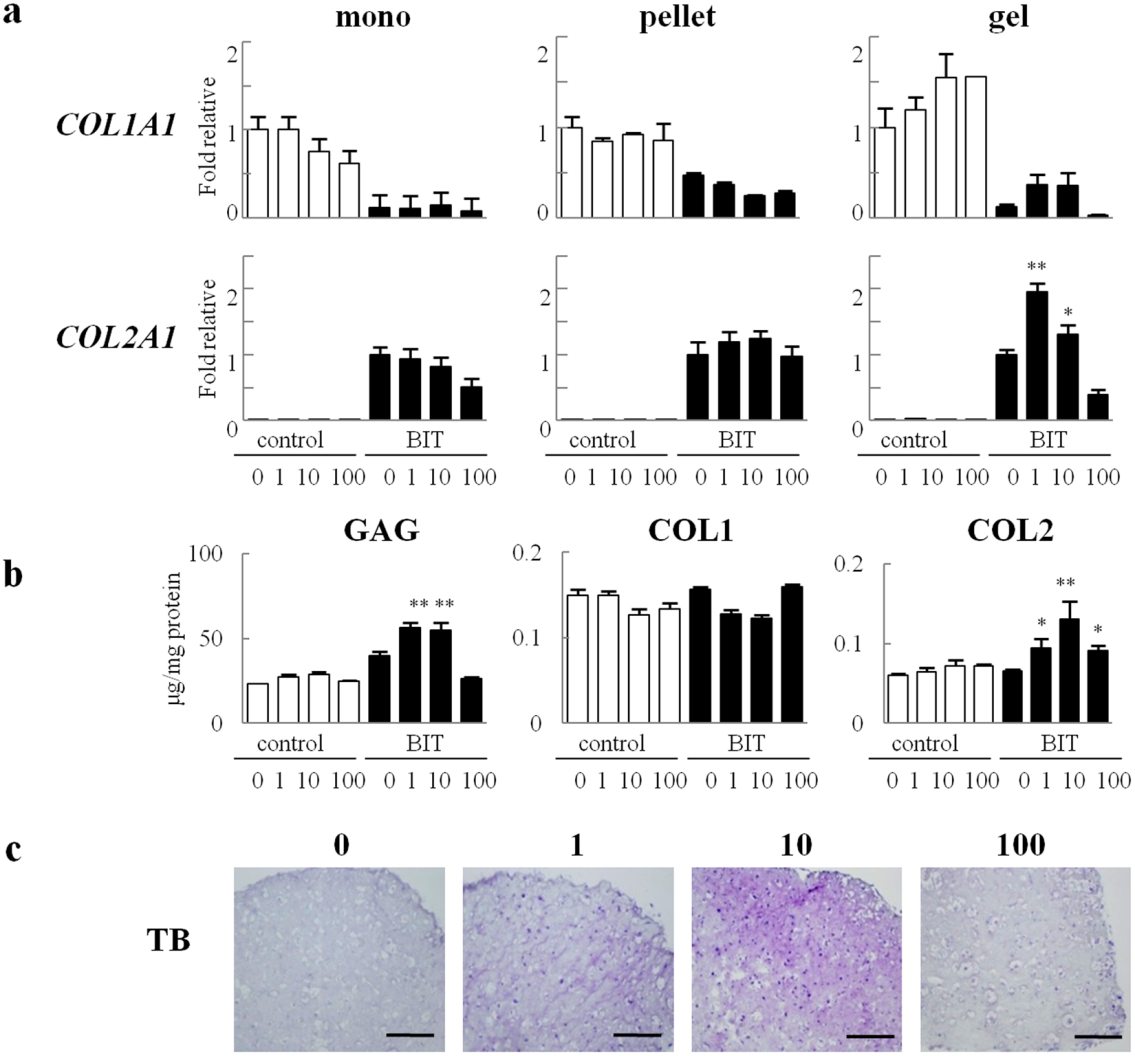
PN-premixed collagen induced chondrogenesis of chondrocytes. (**a**) Gene expression in human chondrocytes cultured on a monolayer (mono) or pellet culture (pellet) with PN addition in culture media was compared to that of chondrocytes embedded in collagen gel pretreated with PN (gel) after 1 week. (**b**,**c**) In the gel culture after 3 weeks, chondrocytes were subjected to GAG, COL1 and COL2 analysis (**b**) and histological analysis by TB (toluidine blue staining) (**c**). Scale bars, 100 µm. Data for (**a**) and (**b**) are expressed as the mean (bars) ± SEM (error bars) for 3 cultures/group. **P < 0.01, vs PN 0 in BIT. cont, control medium. BIT, chondrogenic differentiation medium containing 200 ng/mL BMP-2, 5 µg/mL insulin and 100 nM T3.

We next examined the effect of PN interaction with collagen *in vitro* using cultured chondrocytes. PN was added to media in the presence of a monolayer or 3D culture of chondrocytes in a collagen gel pellet to verify the effect on cartilaginous matrix formation and cartilage regeneration. There was no difference in chondrogenesis following PN addition (Fig. 4a mono and pellet). Conversely, PN mixed in the collagen gel enhanced the expression of COL2A1 (Fig. 4a gel) as well as cartilaginous matrix accumulation, GAG and COL2, at 3 weeks. Chondrocytes cultured in collagen gel with 10 μg/mL PN showed fair differences in protein levels (GAG and COL2) and distinct metachromasia in TB staining (Fig. 4b,c). We also investigated the interaction between PN and hyaluronic acid, which is one of the main extracellular matrices of cartilage. In contrast to PN-premixed collagen gel, PN-premixed in hyaluronic acid gel did not show any influence on chondrogenesis (Fig. S2). The results suggested that PN affects the cartilage maturation distinctively through the interaction with collagen.

### The effect of PN-premixed collagen on cell signaling in chondrocytes

**Figure 5:**
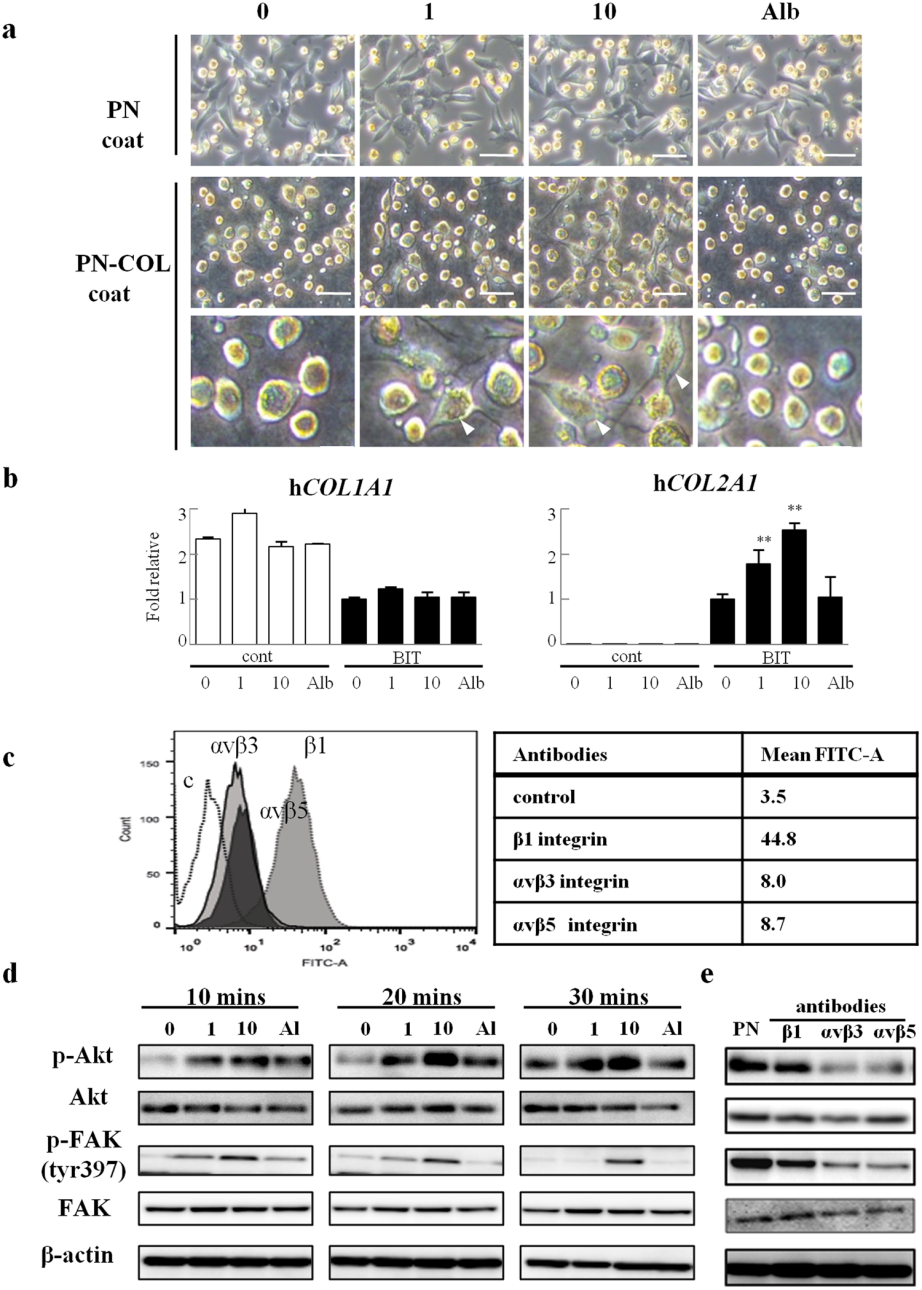
PN-premixed collagen enhanced the Akt/FAK signaling of chondrocytes. (**a**) Human chondrocytes cultured on plates coated with 1 or 10 µg/mL PN (PN coat) or collagen pretreated with 1 or, 10 µg/mL PN (PN-COL coat) after 3 hours. The PN-COL coat could increase the number of adhesive chondrocytes (arrowheads), while the PN coat could not. Scale bars, 20 µm (upper, middle) and 10 µm (lower). (**b**) Gene expression of chondrocytes cultured on PN-COL coat (10 µg/mL) after 1 week. (**c**) Surface expression of αvβ3, αvβ5, or β1 integrins was analyzed by fluorescence-activated cell analysis. Chondrocytes cultured on the PN-COL coat (10 µg/mL) after 1 hour were incubated with mAb against αvβ3, αvβ5, or β1 integrins followed by FITC-labeled goat anti-mouse IgG. The mean fluorescence intensity compared with the control (mAb against IgG1 used as a negative control) was shown in the table. (**d**) Western blot analysis of chondrocytes cultured on PN-COL coat (10 µg/mL) after 10–30 minutes. Alb, bovine serum albumin (10 µg/mL) used as a control for PN. (**e**) Chondrocytes were incubated with mAb against αvβ3, αvβ5, or β1 integrins before being added to a PN-COL coat (10 µg/mL). Western blot analysis of chondrocytes cultured on the PN-COL coat after 30 minutes. PN showed a line of mAb-free chondrocytes cultured on the PN-COL coat. Data for (**b**) are expressed as the mean (bars) ± SEM (error bars) for 3 cultures/group. **P < 0.01, vs PN 0 in BIT. cont, control medium. BIT, chondrogenic differentiation medium containing 200 ng/mL BMP-2, 5 µg/mL insulin and 100 nM T3.

Additionally, we compared the morphology of chondrocytes seeded on a plate coated with PN (Fig. 5a PN coat) or coated with collagen pre-mixed with PN (Fig. 5a PN-COL coat). PN coating did not affect the morphology of chondrocytes. Conversely, chondrocytes on the PN-COL coating showed increased cell adhesion (Fig. 5a arrowheads) and enhanced expression of *COL2A1* (Fig. 5b). These results prompted us to investigate the phosphorylation status of Akt and focal adhesion kinase (FAK) because they are known to play a central role in integrin signaling during collagen interaction and to induce cartilage differentiation by transmitting the extracellular signal of type I and II collagen^20,21^. We analyzed integrin expression of chondrocytes cultured on a PN-COL coat plate using flow cytometry. Chondrocytes cultured on the PN-COL coating expressed the β1, αvβ3, and αvβ5 integrins (Fig. 5c). In addition, chondrocytes cultured on a PN-COL coat plate showed increased Akt and FAK phosphorylation (Fig. 5d). The phosphorylation of chondrocytes to a PN-COL coat plate was inhibited by anti -αvβ3 or anti -αvβ5 integrins mAb. Conversely, the function blocking mAb to β1 integrins did not affect the phosphorylation of chondrocytes (Fig. 5e). These results indicated that PN could biologically work through the interaction with collagen and promote the differentiation of chondrocytes by activating signal transduction mediated by integrins.

### The tissue-engineered cartilage encapsulated with PN-COL could maintain transplant shape

**Figure 6:**
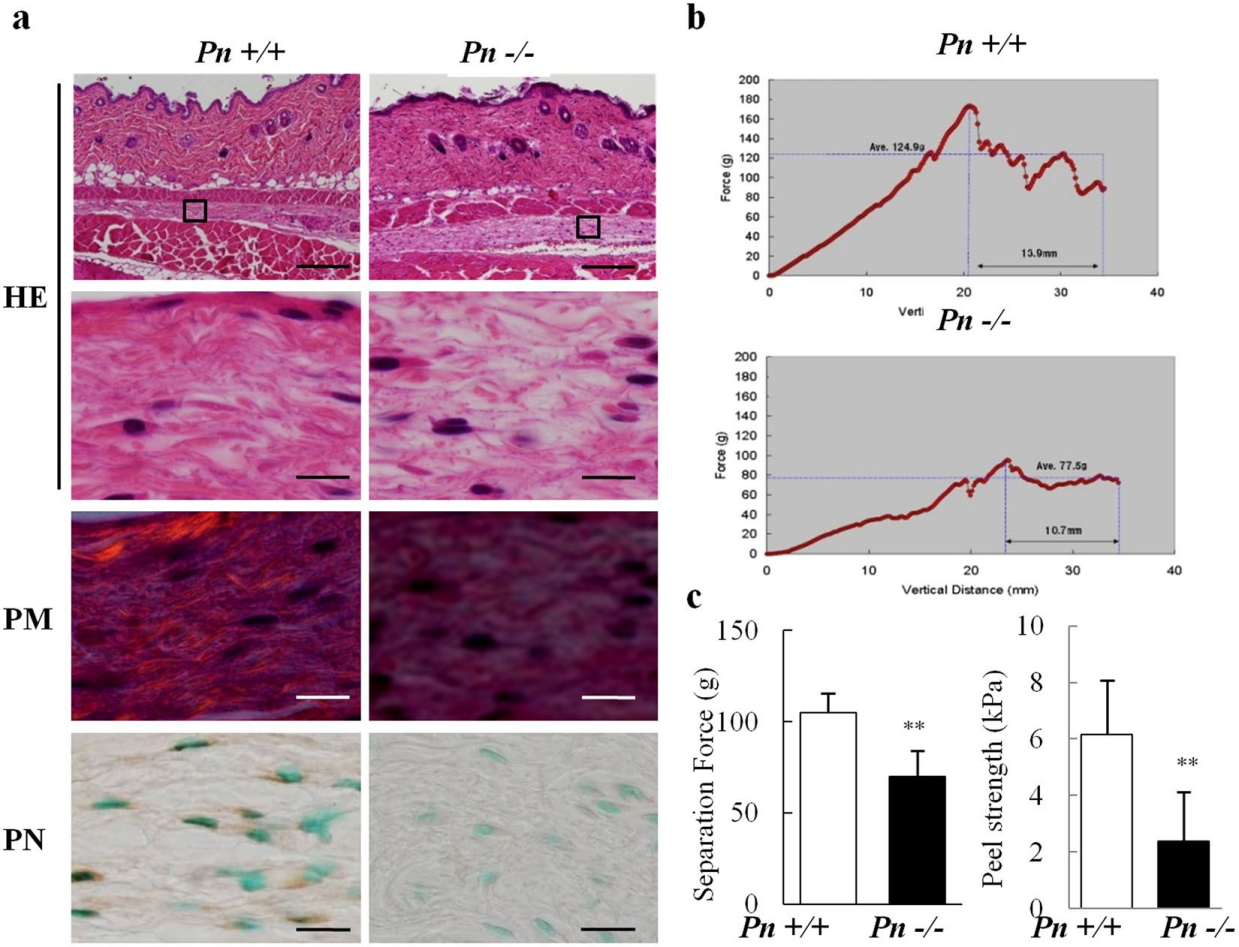
The analysis of *Pn*−/− subcutaneous fibrous tissue. (**a**) Histological analysis of subcutaneous tissues in *Pn*+/+ and *Pn*−/− mice. HE staining, polarizing microscopy (PM) images, and immunohistochemical staining for PN. (**a**) Scale bars: 200 µm(upper) and 20 µm(lower) for HE and 20 µm for PM and PN. (**b**,**c**) Mechanical curve, separation force, or peel stress were measured by peeling upward on subcutaneous fibrous tissue from *Pn*+/+ or *Pn*−/− mice. This experiment was conducted three times and showed similar results. The typical results are shown. Data are expressed as the mean (bars) ± SEM (error bars) from 3 independent experiments/group. **P < 0.01, vs *Pn*+/+ mice.

**Figure 7:**
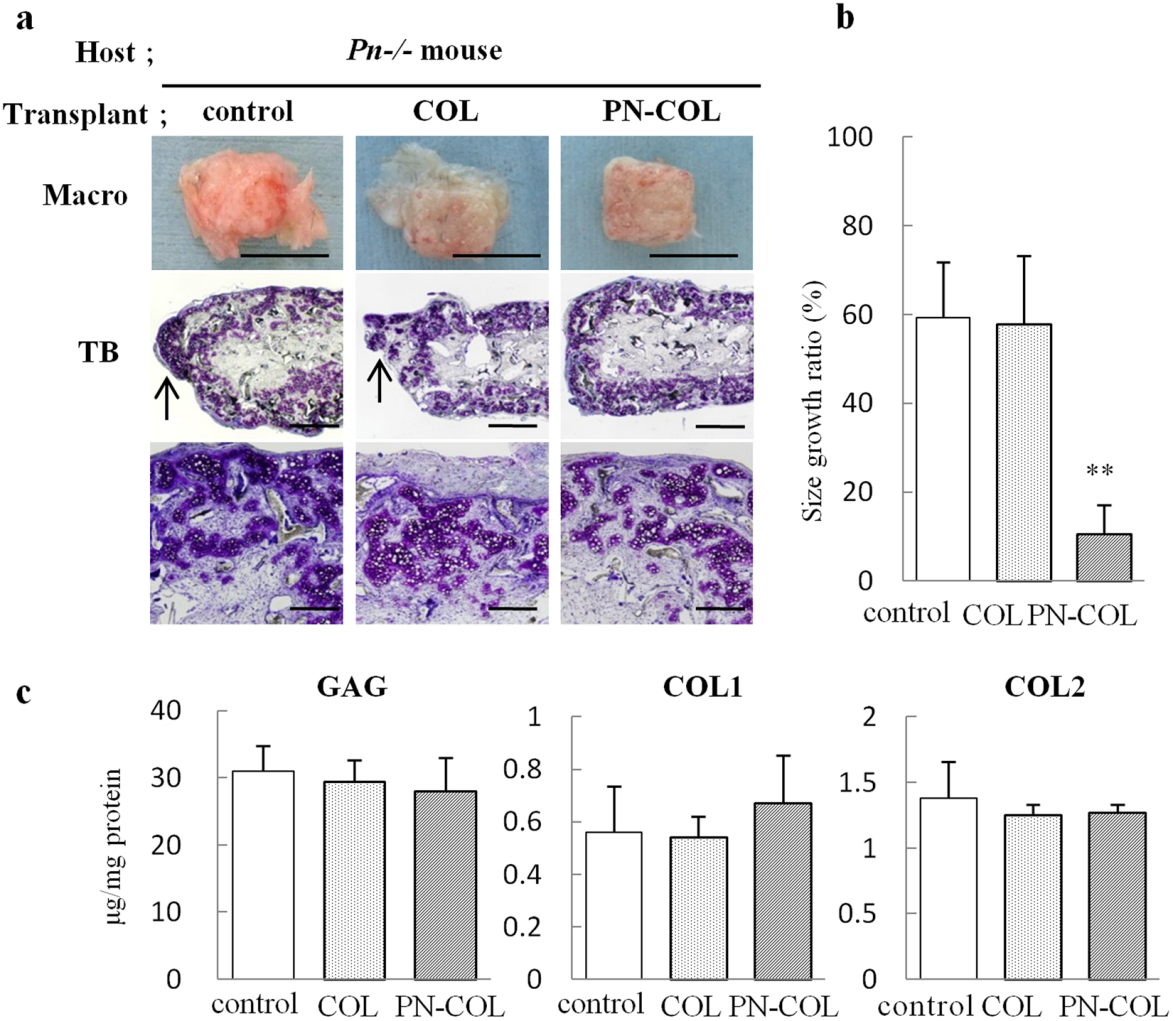
The transplantation of the tissue-engineered cartilage encapsulated with PN-COL into *Pn*−/− mice. (**a**–**c**) Tissue–engineered cartilage constructs, which consisted of *Pn*+/+ auricular chondrocytes (control) encapsulated with collagen (COL) or collagen pretreated with PN (PN-COL), were transplanted into *Pn*−/− mice. Eight weeks after transplantation, the constructs were examined for morphological changes using macroscopic (macro) and histological analysis with TB staining (**a**), size growth ratio (**b**) and accumulation of proteins (**c**). The sites bulging from the scaffold were indicated by arrows (**a**). (**b**) and (**c**) are expressed as the mean (bars) ± SEM (error bars) for 3 implants/group. *P < 0.05, **P < 0.01, vs control. (**a**) Scale bars: 5 mm for macroscopic findings, 1 mm (upper) and 500 µm (lower) for TB (toluidine blue staining).

Last, we determined whether PN could maintain the shape of transplants by increasing the mechanical strength through the promotion of collagen conformation. We examined the subcutaneous fibrous tissue corresponding to the transplantation site of the engineered cartilage to investigate the role of PN in maintaining the mechanical integrity of collagenous tissue *in vivo*. The *Pn*+/+ subcutaneous fibrous tissue consisted of thick conformational fibers emphasized by a polarizing microscope (PM) (Fig. 6a). In contrast, the fibrous tissue of *Pn*−/− mice seemed loose and consisted of thin fibers. The peeling upward test showed that the separation force and peel stress were significantly decreased in the *Pn*−/− mice (Fig. 6b,c). To examine whether PN interaction with collagen can facilitate shape retention in transplants, we transplanted constructs consisting of *Pn*+/+ chondrocytes (Fig. 7a control) and encapsulated with collagen (Fig. 7a COL) or collagen pre-mixed with PN (Fig. 7a PN-COL) into *Pn*−/− mice. Transplants of the control and those encapsulated with COL still exhibited irregular shapes, while those encapsulated with PN-COL maintained their shapes (Fig. 7a,b). In histological findings, the sites bulging from the scaffold which cause the morphological irregularities of transplants consisted of regenerated cartilage tissues (Fig. 7a arrows). There was no difference in the content of GAG, COL1 or COL2 among the transplants (Fig. 7c). We further performed similar experiments using large animals with normal immunity. The transplants made from autologous beagle chondrocytes encapsulated with collagen only (Fig. S3a exterior -) extended out of the original shapes, while those encapsulated with collagen pretreated with PN (Fig. S3a exterior PN-COL) maintained their original shapes (Fig. S3a,b) and had increased rigidity compared to transplants encapsulated with collagen only (Fig. S3d). PN added to the interior of transplants induced the maturation of the cartilage and abundant cartilaginous matrices such as GAG and COL2 (Fig. S3c).

## Discussion

We showed that PN helped the tissue-engineered cartilage to maintain its shape and that it promoted maturation by direct interaction with collagen molecules. PN can bind to other extracellular proteins through each domain: the EMI domain combined with fibronectin^22^ and the FAS1 domain combined with tenascin C^22^ and BMP1^23^. The previous study demonstrated that periostin could directly interact with type I collagen by immunoprecipitation and immunogold transmission electron microscopy experiments, and suggested that periostin could bridge and stabilize adjacent collagen fibrils during fibril fusion^17^. However, the direct binding of PN via specific domain to collagen has never been reported. As shown in our *in vitro* experiments, PN could react with collagen molecules without any extraneous factors, and thick collagen fibers were induced by laterally fusing collagen fibrils with each other, as seen in Fig. 3b. In addition, the EMI domain could directly bind to collagen. The EMI domain was reported to bind with the globular C1q (gC1q) domain^24^, which has a repeating amino acid sequence of Gly-Pro-X that is also characteristically observed in collagen^25,26^. Considering that this characteristic structure is also present in collagen, it was reasonable that PN exhibited a high affinity for COL1 through the EMI domain.

Moreover, we revealed that PN can enhance the extracellular signal for chondrocytes when mixed with collagen. There have been many reports that PN is involved in various biological processes, including wound healing^27^, tumorigenesis^28^, and recovery in myocardial infarction^18^. Signaling from ECM affects the behavior of cells. During tumor progression, binding to fibrillary collagen via the collagen receptor inhibits the proliferation of tumor cells through the regulation of integrin clustering and the activation of FAK. In contrast, binding to the monomeric form of fibrillary collagen increases the contact with collagen mediated by α2β1 integrin and the proliferation of tumor cells^29^. Similarly, changes in the ECM structure induced by PN can also enhance signal transduction^30^. It has been reported that mesenchymal cells cultured in PN-treated collagen matrix can induce cell invasion through integrin signaling^31^, and the adhesion of epithelial carcinoma cells is suppressed by the antibodies against αvβ3 or αvβ5 integrin^32^. In those adhered cells, αvβ3 integrin localized to the focal adhesion plaques. The formation of focal adhesion plaque requires tension or rigidity of the substrate^33^. For chondrocytes, FAK and Akt activation through integrin can stimulate cell differentiation^20^. Our data show that the addition of PN to culture medium has no effect, while a collagen gel pre-mixed with PN could enhance Akt and FAK signaling and induce the production of cartilaginous matrix by chondrocytes. We hypothesizes that conformational changes in collagen induced by periostin can alter the cell-substratum adhesion and affect the signal transduction in chondrocytes. On the other hand, it cannot be denied that the physical changes (changes of mechanical strength) of collagen matrix induced by periostin regulates the mechanotransduction and influences the signal transduction of chondrocytes. The fate of cellular differentiation is regulated by the elasticity of the substrate^34^, and mesenchymal stem cells cultured on a hard substrate activates FAK mediated by integrins^35^. We need further consideration to the possibility that mechanical changes in the collagen scaffold induced by periostin will regulate signal transduction.

In our past study, we investigated the existence of host- and transplant-derived cells in tissue-engineered cartilage constructs transplanted subcutaneously using *EGFP* transgenic mice as hosts^36^. The transplant-derived cells mainly constituted the areas of regenerated cartilage in constructs, and were seldom found in the surrounding fibrous tissues, while most of the host-derived cells detected by GFP expression were present around constructs, and a part of the host-derived cells that infiltrated into constructs consisted of F4/80-positive macrophages. These results indicated that surrounding fibrous tissues were predominantly composed of host-derived fibroblasts and partly contained host-derived macrophages. In this study, the constructs transplanted into *Pn*−/− mice exhibited an irregular shape because of weakened surrounding tissue devoid of PN, which could not prevent physical outflow from the scaffold. Although this outgrowth of the transplant may also be attributable to an external fibrous tissue, our histological findings (e.g., Fig. 7a) showed that swelling tissues out of the scaffold were mainly composed of regenerated cartilage tissues, suggesting that the morphological irregularities of transplants were induced by physical outflow of regenerated cartilage tissue from the scaffold but not by hypertrophy of the external fibrous tissue.

PN deficiency in the host also affects tumor morphology in a tumor-bearing model^37^. In *Pn*+/+ mice, PN was expressed in fibrous tissues surrounding tumors and could suppress tumor spread. A tumor formed in *Pn*−/− mice had a capsule in which fibrous tissue was poorly formed and showed a trend toward expansion. When transplanting the tissue-engineered cartilage, we observed that PN was also effective at maintaining the shape of the transplants by improving the strength of the fibrous tissue between the host and the transplants. In addition, applying PN-COL around transplants could improve the mechanical strength of the surrounding layer and increase the rigidity of actual cartilage constructs which could help the shape retention of transplants, while it did not affect the maturation of transplants.

Cartilage diseases are diverse, and they are not limited to local defects of articular cartilage but also include facial cartilage defects. Repairing local defects of articular cartilage supported by hard tissue, it is desirable to avoid fibrous tissue encapsulating the transplants to promote integration with surrounding cartilage. On the other hand, repairing defects of facial cartilage surrounded by soft tissue may be desirable for maintaining the transplants in the surrounding fibrous tissue. We produced the tissue-engineered cartilage with 3D morphology and mechanical strength using a biodegradable polymer scaffold, and already applied it to clinical research for patients with nasal deformities associated with cleft lips and palates^38^. In such a subcutaneous transplantation of an engineered cartilage, the surrounding tissue helps the shape retention of transplants and has profound significance.

**Figure 8:**
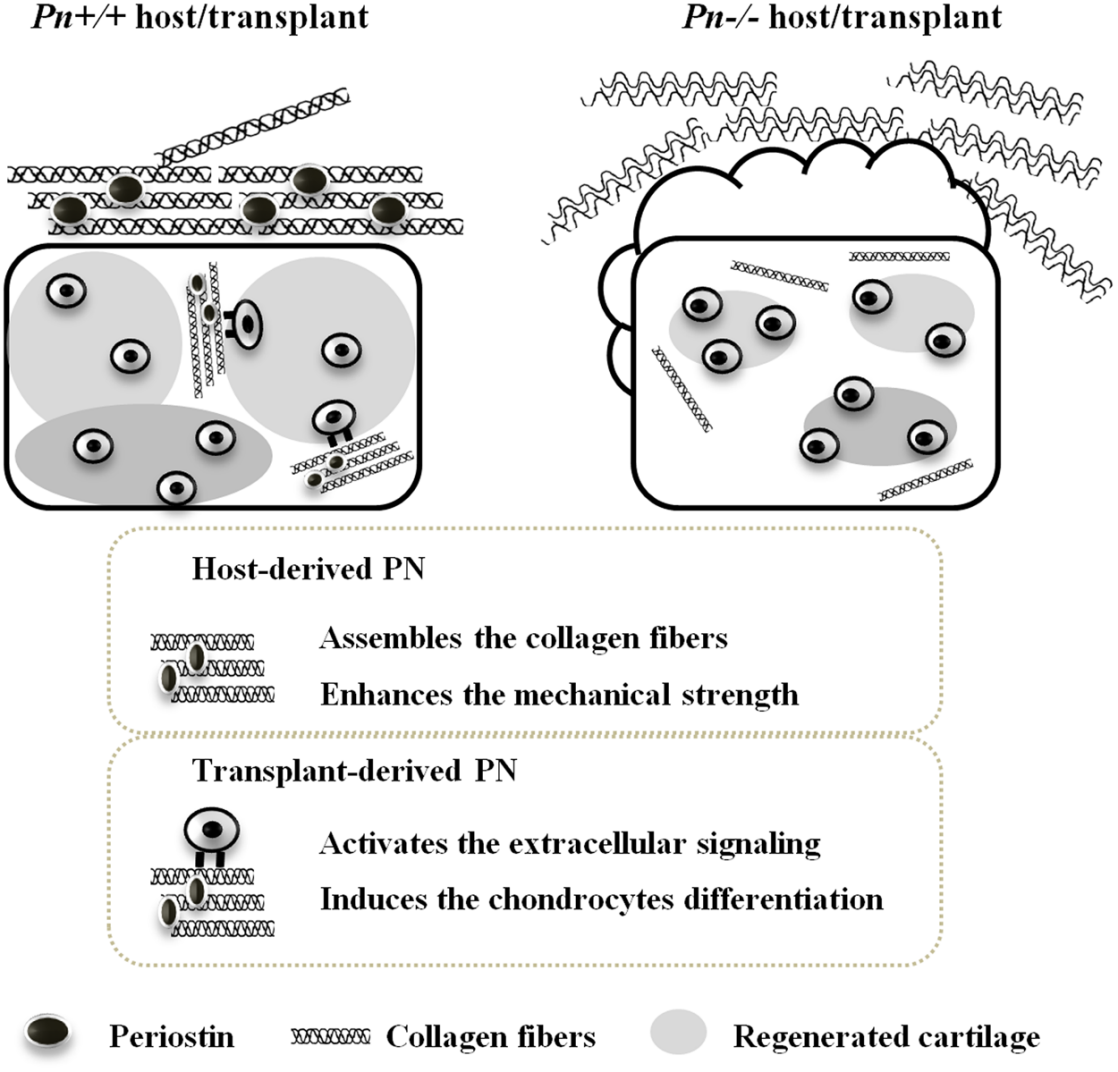
A schematic representation of the role of PN in the shape retention and the maturation of the tissue-engineered cartilage. PN derived from a host contributes to the shape retention of the tissue-engineered cartilage construct, and PN derived from a transplant contributes to its maturation. These two different effects are induced by the function of PN, which interacts with type I collagen (COL1) through the EMI domain and improves the collagen conformation. The host-derived PN co-localizes with COL1 in the surrounding fibrous tissues. The PN-mediated collagen structure enhances the mechanical strength of those tissues. The transplant-derived PN co-localizes with COL1 in interstitial fibrous tissues. The PN-mediated collagen structure activates the extracellular signaling mediated by integrins and induces the production of cartilaginous matrix by chondrocytes.

We performed the first successful tissue-engineered cartilage transplantation regulating the morphological changes after transplantation and succeeded in promoting cartilage maturation by utilizing the function of PN. PN derived from a host could be critical to maintaining the shape of the regenerative cartilage, and PN derived from a transplant could advance its maturation (Fig. 8). The unique function of PN inducing the high-order structure of collagen might produce these two different effects in regenerative cartilage. The limitations of this study are that (1) the interactions between periostin and collagen and the periostin-collagen complex and integrins have not been elucidated clearly, and (2) the mechanical properties of the surrounding tissue were not analyzed separately, because it is difficult to isolate those tissues. Nevertheless, this study has uncovered a new paradigm for controlling the 3D shape of regenerative tissue after transplantation, and these newly discovered effects of PN could be useful in clinical practice.

## Materials and Methods

### Cell culture

All materials are described in SI. Human cartilage was obtained after informed consent from the remnant auricular cartilage of microtia patients. All procedures for the present experiments were approved by the ethics committee or institutional committee for animal research of the University of Tokyo Hospital (ethics permission #622), and the experiments were conducted according to the principles expressed in the Declaration of Helsinki. The isolation and cell culture of human and mouse auricular cartilages were conducted as previously described^36^. Medium, containing 200 ng/mL BMP-2, 5 µg/mL insulin and 100 nM T3 was used as the chondrogenic medium (described as BIT). For comparison, non-supplemented DMEM/F12 was used as the control medium. Human auricular chondrocytes were cultured on a monolayer (mono) or pellet culture (pellet) similar to as previous studies^36,39^, with human recombinant PN added at 1, 10, and 100 µg/mL to the chondrogenic medium for 1 week. To compare the addition of PN to the culture media, chondrocytes were cultured in a collagen gel premixed with recombinant PN (1, 10, 100 µg/mL) (gel). Human auricular chondrocytes were cultured on plates coated with 1 and 10 µg/mL PN (PN coat) or collagen with 1 and 10 µg/mL PN (PN-COL coat) for 1 week. Chondrocytes were also cultured on plates coated with 10 µg/mL albumin or collagen with 10 µg/mL albumin for 1 week as the control. Gene expression in human auricular chondrocytes cultured as a monolayer (mono) or pellet culture (pellet) with PN added to the chondrogenic medium was compared to those embedded in a collagen gel pretreated PN (gel) after 1 week.

### Production and transplantation of tissue-engineered cartilage

*Periostin*−/− (*Pn*−/−) mice were originally produced by Shimazaki, M.^18^ In accordance with the published method, we produced the tissue-engineered cartilage^36^. To briefly describe how the tissue-engineered cartilage was made, cells were suspended in 1% atelocollagen gel (Koken Co., Ltd), and the cell suspension was applied to a 5 × 5 × 3 mm biodegradable poly-L-lactic acid (PLLA) scaffold (GC R&D Center, Tokyo, Japan). The cell suspension was applied to the scaffold at 2.5 × 10^7^ cells/250 μL. We made transplants of tissue-engineered cartilage originating from *periostin*−/− (*Pn*−/−) or *periostin*+/+ (*Pn*+/+) C57BL/6 J mice (described as Transplant: *Pn*+/+ or *Pn*−/−). Regarding the transplantation procedure, 6-week-old male *Pn*−/− or *Pn*+/+ mice were anesthetized by intraperitoneal injection of sodium pentobarbital (50 mg / kg). A small incision was made on the back at the midline, and two types of transplants (Transplant: *Pn*+/+ or *Pn*−/−) were subcutaneously transplanted into each animal (Host: *Pn*+/+ or *Pn*−/− mice). Eight weeks after the operation, the harvested transplants were assessed by histological and immunohistochemical analyses. The area of tissue-engineered cartilage extending out of the PLLA scaffolds was measured at 8 weeks. The ratio of areas of regenerative cartilage extending out of PLLA scaffolds compared to those of the original areas (size growth ratio) was measured. Data are expressed as the mean (bars) ± SEM (error bars) of the size growth ratio in 3 images from three independent experiments. Some constructs were encapsulated with collagen (COL) or collagen pretreated with 10 µg/mL periostin (PN-COL). This experiment was performed 5 times using 40 donor mice and 20 host mice. Three typical results from 5 experiments were used as the comparison group.

### Analysis of collagen with added PN

Recombinant PN (1, 10, or 100 µg/mL) was added to a 0.8% atelocollagen solution. The 200 µL mixture was poured into a cloning ring (4 mm diameter, 1 cm height). Each mixture was incubated at 37 °C for 2 hours. After the collagen was gelled, the gelation of the collagen with added PN (1, 10, or 100 µg/mL) was compared to that of the collagen without PN (0 µg/mL). Morphological and ultrastructural changes were observed after removal from the ring.

### Purification of the EMI domain deletion form of PN and no deletion form of PN

The expression vectors for HA-tagged PN (PN-HA) and its deletion form (*Δ*EMI-HA) were based on the pCAGIPuro backbone, which was provided by Dr. A. Kudo^16^. Plasmid DNAs were chemically transformed into One Shot TOP10 cells according to the manufacturer’s instructions. Bacteria were selected with 100 µg/mL ampicillin and colonies were screened for the correct insert orientation by colony PCR. Plasmid DNA was purified from the cultured bacteria using a QIAPrep Spin Miniprep Kit. Recombinant PN-HA and *Δ*EMI-HA were produced by the Freestyle 293 expression system. Suspension Freestyle 293-F cells were grown in FreeStyle 293 Expression Medium in shaker flasks at 37 °C with 8% CO_2_. The cells were transfected on a 30 mL plate with 30 µg of DNA and 40 µL of 293Fectin at a cell density of 1.1 × 10^6^ / mL. Forty-eight hours later, the lysates were centrifuged at 1,000 × *g* for 5 min, and supernatants were incubated for 1 hour with anti-HA antibody-conjugated agarose at 4 °C. The bound proteins were eluted with SDS sample buffer, and PN-HA and *Δ*EMI-HA proteins were purified.

### Mechanical properties of subcutaneous tissues

Subcutaneous tissue 2 × 4 cm in size, which consisted of skin tissue, connective tissue, and muscle tissue, was excised from the back of the 6-week-old male *Pn*−/− or *Pn*+/+ mice. With the skin surface up, the sample was fixed to the pedestal at the lower surface and lifted upward at a constant speed (45 mm/min). The mechanical curve and peel stress were measured by upward peeling of the subcutaneous fibrous tissue from the *Pn*+/+ or *Pn*−/− mice. This peeling test method was conducted in accordance with the JIS standard method (JIS K6854-1). The peel strength was calculated by dividing the mean load by the sample area.

### Histology, immunohistochemical staining, and ultrastructural studies

Each sample was fixed in 4% paraformaldehyde, embedded in paraffin, and cut into 8 µm sections. The sections were stained with toluidine blue to detect proteoglycan as well as with hematoxylin and eosin (H–E staining). The sections were immunohistochemically stained for periostin (PN), type I collagen (COL1), and type II collagen (COL2) based on previous studies^36^ and the manufacturers’ instructions and observed using an optical microscope (Olympus DP 70, Tokyo, Japan). For the ultrastructural observation of the collagen gel, each sample was immersed in a mixture of 2% paraformaldehyde and 2.5% glutaraldehyde. Employing an Ultracut UCT microtome (Leika, Wien, Austria), the Poly/Bed-embedded specimens were sliced into 0.5 µm sections. These sections were observed using a transmission electron microscope (TEM; H-7100, Hitachi, Tokyo, Japan).

### RNA isolation and real-time RT-PCR

The total RNA from cells cultured for 7 days was isolated with Isogen and reverse-transcribed with PrimeScript reverse transcriptase and random hexamers. Gene expression was detected by real-time qPCR using the standard SYBR green method with an ABI 7500 Real-Time PCR System (Applied BioSystems, CA, USA). The sequences of the primers are described in SI.

### Biochemical measurement of Glycosaminoglycan (GAG) and Type I and Type II collagens

The sulfated GAG content of the tissue-engineered cartilage was measured using the alcian blue-binding assay, and the collagen proteins of the tissue-engineered cartilage were quantified by ELISA using a human Type 1, and 2 Collagen Detection Kit.

### Western blot analysis

Human auricular chondrocytes were collected after culturing on collagen gel with 10 µg/mL periostin for 10, 20, or 30 minutes. Samples were prepared using M-PER (Pierce Biotechnology, Lockford, IL) supplemented with Na_3_VO_4_ (2 mM), NaF (10 mM), and aprotinin (10 µg/mL). An equal amount (20 µg) of protein was subjected to SDS-PAGE and transferred onto PVDF membranes. Rabbit polyclonal antibodies against Akt, FAK, phospho-Akt, phosphor-FAK, and α-actin were used as the primary antibodies. The membrane was incubated with HRP-conjugated secondary antibody. Immunoreactive proteins were visualized by ECL.

### Binding assay

We coated 96-well plates with 2.5 µg/mL PN, fibronectin, hyaluronic acid, COL1, COL2, or BSA. After blocking with blocking buffer (Tris-buffered saline containing 3% BSA, and 0.05% Tween-20), the indicated concentrations of biotinylated PN (PN-HA) or the EMI domain deletion form of PN (*Δ*EMI-HA) were added to binding buffer (Tris-buffered saline containing 1% BSA, 0.05% Tween-20, and 10 mmol/L CaCl_2_) and incubated overnight at 4 °C. After extensive washing, streptavidin-conjugated horseradish peroxidase was incubated, and signals were detected by OPD. The spectrophotometric absorbance was measured at 490 nm. The data were corrected by evaluating the degree of biotinylation of each protein.

### Statistics

All data are expressed ± S.E. Comparisons of the means between two independent groups were performed by t-test. Other comparisons among the groups were performed by ANOVA, and the significance of differences was determined by post hoc testing using Bonferroni correction.

## Electronic supplementary material

Supplementary inforamation
