# Supplementary material for: Periostin contributes to the maturation and shape retention of tissue-engineered cartilage
Source: Sci Rep. 2018 Jul 25;8:11210. doi: 10.1038/s41598-018-29228-6 (PMC6060118; doi:10.1038/s41598-018-29228-6)
Supplement: Supplementary file 1 — Supplementary inforamation [file 41598_2018_29228_MOESM1_ESM.pdf]

Periostin contributes to the maturation and shape retention of tissue-engineered cartilage

Ryoko Inaki[1,2], Yuko Fujihara[1], Akira Kudo[3], Masaki Misawa[4], Atsuhiko Hikita[2], Tsuyoshi Takato[5] and Kazuto Hoshi[1,2]

1. Department of Oral-maxillofacial Surgery, Dentistry and Orthodontics, The University of Tokyo Hospital, Tokyo, Japan,
2. Division of Tissue Engineering, The University of Tokyo Hospital, Tokyo, Japan
3. Department of Biological Information, Tokyo Institute of Technology, Tokyo, Japan
4. Institute of Human Science and Biomedical Engineering, National Institute of Advanced Industrial Science and Technology, Tsukuba, Ibaraki, Japan
5. JR Tokyo General Hospital, Tokyo, Japan

Correspondence to:
Kazuto Hoshi, M.D., Ph.D.,
Department of Oral-maxillofacial Surgery, Dentistry and Orthodontics, The University of Tokyo Hospital,
Hongo 7-3-1, Bunkyo-ku, Tokyo 113-0033, Japan
e-mail: pochi-tky@umin.net
TEL&FAX +81-3-5800-9891

(Author information)

Correspondence and requests for materials should be addressed to H.K. (pochi-tky@umin.net).

Supporting Information

SI Methods

Materials

Dulbecco's modified Eagle's medium (DMEM), Dulbecco's modified Eagle's medium/nutrient Mixture F-12 Ham (DMEM/F12), penicillin–streptomycin solution, and trypsin–EDTA solution were purchased from the Sigma Chemical Co. (MO, USA). Collagenase from *Clostridium histolyticum* was from Wako Pure Chemical Industries (Osaka, Japan), insulin was from MP Biomedicals (CA, USA), FGF-2 was from Kaken Pharmaceutical Co., Ltd. (Tokyo, Japan), BMP-2 was from Astellas Pharmaceutical Co., Ltd. (Tokyo, Japan), and l-3, 5, 3′- triiodothyronine (T3) was from Bioscience (CA, USA). Other materials included a 3% atelocollagen implant from Koken Co., Ltd. (Tokyo, Japan), PLLA porous scaffolds from KRI (Kyoto, Japan), recombinant human / mouse periostin from R&D System, Inc. (Tokyo, Japan), anti-human Akt / phosphor-Akt and anti-human FAK / phosphor-FAK (tyr397) from CST, Inc. (MA, USA), anti-rabbit IgG, HRP-linked whole antibody, and ECL plus from GE Healthcare, Ltd. (Buckinghamshire, UK), anti-periostin(PN) antibody from Abcam, Inc. (MA, USA), and anti-type-I collagen(COL1) antibody and anti-type-II collagen(COL2) antibody from LSL, Inc. (Japan). The biotinylated secondary antibody, Vectastain Elite ABC Kit and Peroxidase Substrate kit DAB were all obtained from Vector Laboratories (CA, USA). Fibronectin (CHEMICON International, Temecula, Calif), COL1 and COL2 (MORINAGA, Yokohama, Japan), and BSA (Wako, Osaka, Japan) were also obtained. The alcian blue binding assay was from Wieslab AB (Lund, Sweden), and theType I, II Collagen ELISA Kit was from Chondrex Inc. (WA, USA). One Shot TOP10 cells, Freestyle 293-F cells, FreeStyle 293 Expression Medium, and 293Fectin were from Invitrogen, the QIAPrep Spin Miniprep Kit was from Qiagen (Hilden, Germany), and the anti-HA antibody-conjugated agarose was from Sigma-Aldrich.

RT-PCR

Total RNA was isolated from cells cultured for 7 days. The sequences of the primers were 5′- ATTCCAGTTCGAGTATGGCG -3′ and 5′- CGACAGTGACGCTGTAGGTG -3′ for human type I collagen αI chain (COL1A1), 5′- TTCAGCTATGGAGATGACAATC -3′ and 5′- AGAGTCCTAGAGTGACTGAG -3′ for human type II collagen αI chain (COL2A1), 5′- GGGCCAGAAAAACAAACTGA -3′ and 5′- ATCACCTTGTGGACCTCTGG -3′ for human PERIOSTIN, 5′- GAAGGTGAAGGTCGGAGTCA -3′ and 5′- GAAGATGGTGATGGGATTTC -3′ for human glyceraldehyde-3-phosphate dehydrogenase(GAPDH), 5′- GAAACCCGAGGTATGCTTGA -3′ and 5′- GGGTCCCTCGACTCCTACAT -3′ for mouse Col1A1, 5′- GGCAAAGATGGCTCTAATGG -3′ and 5′- CGTCGTGCTGTCTCAAGGTA -3′ for mouse Col2A1, 5′- GAACGAATCATTACAGGTCC -3′ and 5′- GGAGACCTCTTTTTGCAAGA -3′ for mouse Periostin, and 5′- AACTTTGGCATTGTGGAAGG -3′ and 5′- ACACATTGGGGGTAGGAACA -3′ for mouse Gapdh. All the primers have been confirmed to be specific to only human or mouse genes. The transcript levels were normalized to that of GAPDH.

Canine model

We conducted canine autologous chondrocyte transplants using a biodegradable poly-L-lactic acid (PLLA) scaffold using the fabrication method of tissue-engineered cartilage for the mouse. Canine auricular chondrocytes were retained in the scaffold with collagen (interior -) or those with 10 μg/mL PN added (interior +PN). After the constructs were encapsulated with collagen pretreated with 10 μg/mL PN (exterior PN-COL) or not (exterior -), they were subcutaneously transplanted on the back of the canine under anesthesia. After 2 months, we harvested the transplants and evaluated them by histological and biochemical analyses and assessment of mechanical properties. The mechanical properties of the transplants were measured using a Venustron tactile sensor (Axiom, Fukushima, Japan). Young's modulus was calculated according to compression load and frequency decrease using Venus 42 software (Axiom) based on a previous report[39,]. This experiment was performed 3 times using 3 beagles.

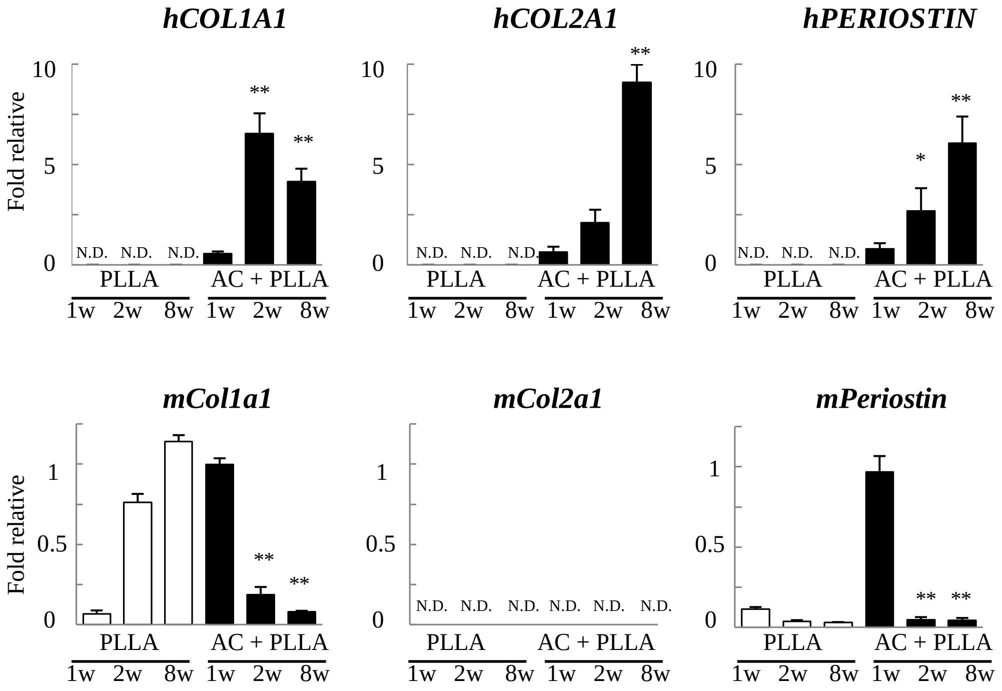

Fig. S1. Time course changes in periostin expression in host-derived or transplant-derived cells.

Time course changes in periostin expression in tissue-engineered constructs consisting of human auricular chondrocytes and PLLA scaffold (AC+PLLA) and PLLA scaffolds without cells (PLLA). Data were expressed as the mean (bars) ± SEM (error bars) of the relative ratio of AC+PLLA at 1 week for 3 transplants/group. **: $P<0.01$, vs AC+PLLA, 1w

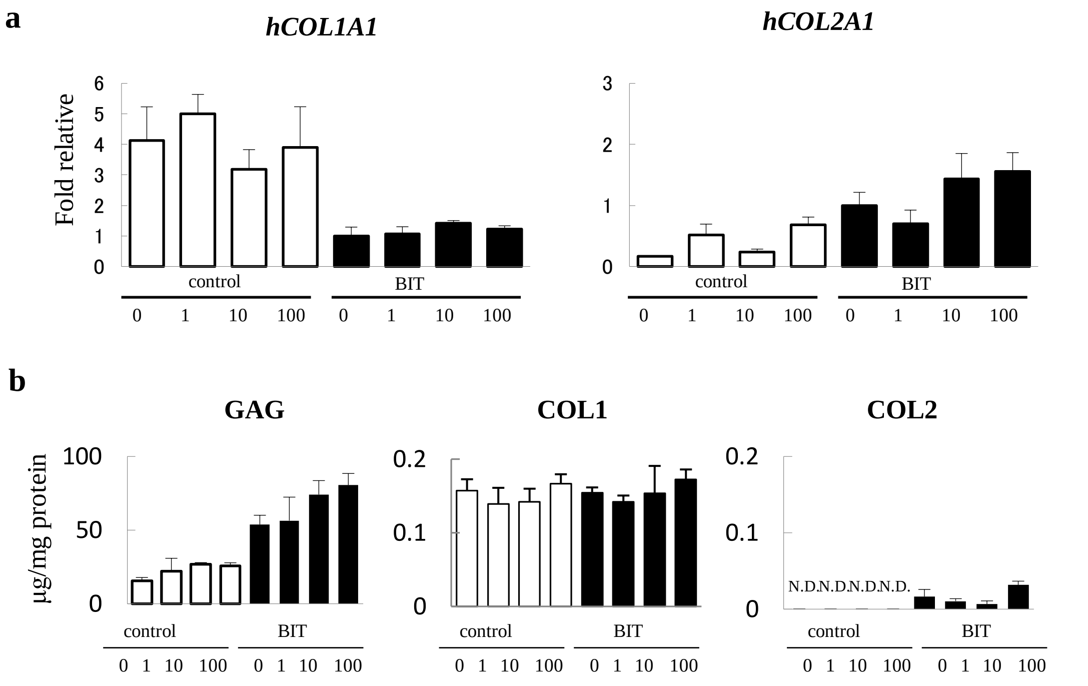

Fig. S2. Analysis of the chondrocytes embedded in hyaluronic acid gel premixed with PN. **a,** Gene expression in human chondrocytes cultured in hyaluronic acid gel with 0, 1, 10, or 100 μg/mL PN after 1 week. **b,** Quantification of the GAG, COL1, and COL2 proteins in the PN-premixed hyaluronic acid gel culture for 3 weeks. Data were expressed as the mean (bars) ± SEM (error bars) of the relative ratio for 3 cultures/group. **: P<0.01, vs PN 0 in BIT. control, control medium. BIT, chondrogenic differentiation medium.

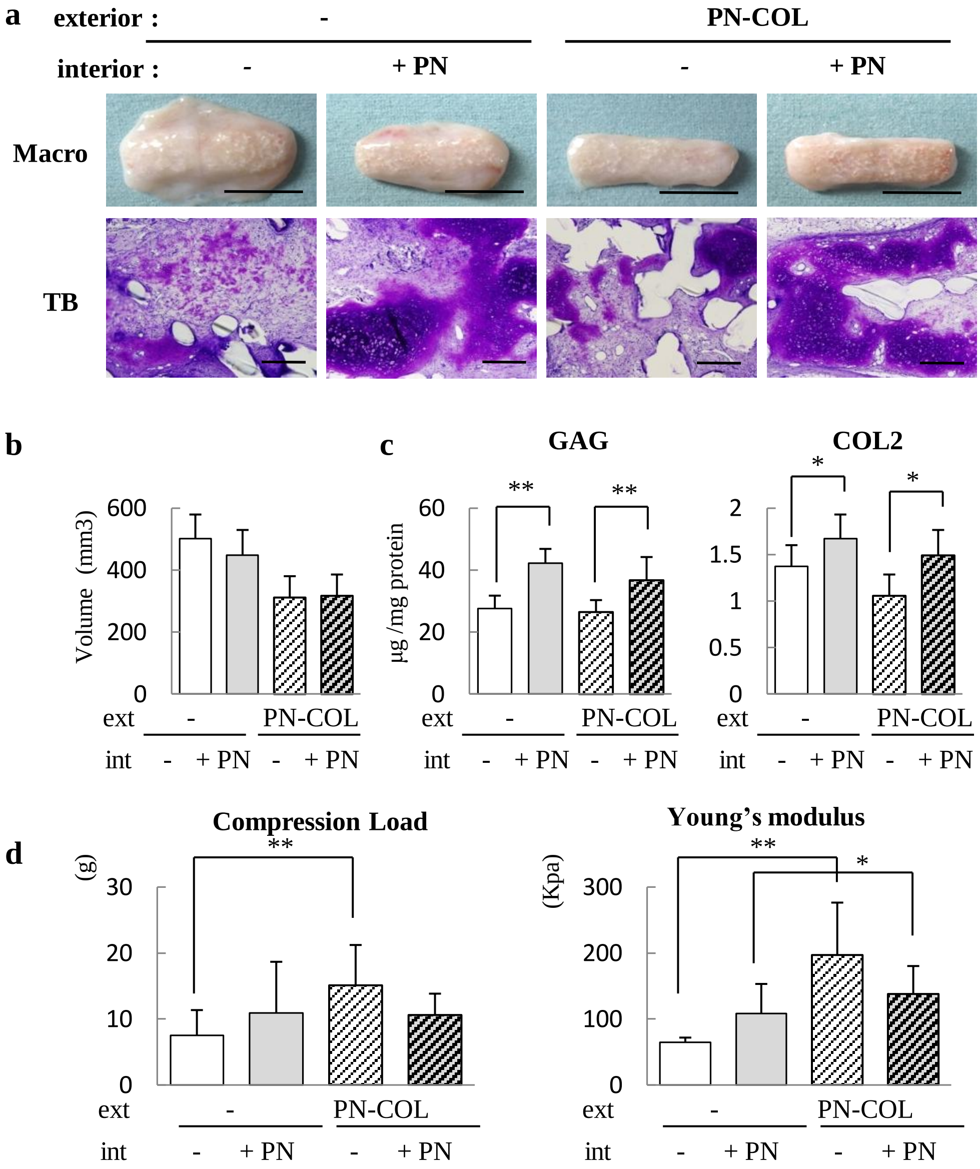

Fig. S3. Morphological and histological features, protein expression, and mechanical properties of tissue-engineered cartilage consisting of canine auricular chondrocytes. The constructs consisted of canine auricular chondrocytes and collagen (int: -) or those with PN-premixed collagen added interior (int: +PN), and encapsulated with collagen pretreated with PN (ext: PN-COL) or not (ext: -). **a,** Morphological features *(upper photos*) and toluidine blue staining (*bottom photos*). **b,** The volume that overflowed from the default size of the scaffold of each construct. **c,** The content of GAG and COL2 was analyzed. **d,** Young's modulus was calculated according to the compression load and frequency decrease using a Venustron tactile sensor. Data for **b – d** are expressed as the mean (bars) ± SEM (error bars) for 3 implants/group. *: P<0.05, **: P<0.01,ext PN-COL vs ext - (**b, c**), int + PN vs int - (**d**). **a**, Scale bars, 5 mm for macroscopic findings and 500 μm for TB. Macro, macroscopic findings. TB, toluidine blue staining.
